# A Single Dose of Atorvastatin Applied Acutely after Spinal Cord Injury Suppresses Inflammation, Apoptosis, and Promotes Axon Outgrowth, Which Might Be Essential for Favorable Functional Outcome

**DOI:** 10.3390/ijms19041106

**Published:** 2018-04-07

**Authors:** Katarina Bimbova, Maria Bacova, Alexandra Kisucka, Jaroslav Pavel, Jan Galik, Peter Zavacky, Martin Marsala, Andrea Stropkovska, Jana Fedorova, Stefania Papcunova, Jana Jachova, Nadezda Lukacova

**Affiliations:** 1 Institute of Neurobiology of Biomedical Research Centre of Slovak Academy of Sciences, Soltesovej 4,6, 040 01 Kosice, Slovakia; bimbova@saske.sk (K.B.); bacova@saske.sk (M.B.); kisucka@saske.sk (A.K.); pavel@saske.sk (J.P.); galik@saske.sk (J.G.); mmarsala@ucsd.edu (M.M.); stropkovska@saske.sk (A.S.); jfedorova@saske.sk (J.F.); gedrova@saske.sk (S.P.); jachova@saske.sk (J.J.); 2 1st Department of Surgery, Louis Pasteur University Hospital, Faculty of Medicine University of Pavol Jozef Safarik, Trieda SNP 1, 041 66 Kosice, Slovakia; peterzavacky963@gmail.com; 3 Neuroregeneration Laboratory, Department of Anesthesiology, University of California, 9500 Gilman Drive, San Diego, CA 92092-0100, USA

**Keywords:** spinal cord compression, inflammatory response, Atorvastatin, caspase-3, macrophages, Gap43, neurofilaments, gene expression

## Abstract

The aim of our study was to limit the inflammatory response after a spinal cord injury (SCI) using Atorvastatin (ATR), a potent inhibitor of cholesterol biosynthesis. Adult Wistar rats were divided into five experimental groups: one control group, two Th9 compression (40 g/15 min) groups, and two Th9 compression + ATR (5 mg/kg, i.p.) groups. The animals survived one day and six weeks. ATR applied in a single dose immediately post-SCI strongly reduced IL-1β release at 4 and 24 h and considerably reduced the activation of resident cells at one day post-injury. Acute ATR treatment effectively prevented the excessive infiltration of destructive M1 macrophages cranially, at the lesion site, and caudally (by 66%, 62%, and 52%, respectively) one day post-injury, whereas the infiltration of beneficial M2 macrophages was less affected (by 27%, 41%, and 16%). In addition, at the same time point, ATR visibly decreased caspase-3 cleavage in neurons, astrocytes, and oligodendrocytes. Six weeks post-SCI, ATR increased the expression of neurofilaments in the dorsolateral columns and Gap43-positive fibers in the lateral columns around the epicenter, and from day 30 to 42, significantly improved the motor activity of the hindlimbs. We suggest that early modulation of the inflammatory response via effects on the M1/M2 macrophages and the inhibition of caspase-3 expression could be crucial for the functional outcome.

## 1. Introduction

Spinal cord injury (SCI) is one of the most devastating and complex clinical conditions, often leading to irreversible neurological deficits. The complex pathophysiology of a SCI, consisting of primary and secondary mechanisms, may explain the difficulty of finding a suitable therapy [1]. SCI is caused by two distinct events, which follow a somewhat overlapping temporal sequence: the acute (seconds to minutes after the injury) phase, the secondary (minutes to weeks after the injury) phase, and the chronic (months to years after the injury) phase [2,3]. In the acute phase, due to the direct mechanical insult, the spinal tissue undergoes a cascade of events followed by secondary damage affecting intact, neighboring tissue [4]. Inflammatory response is one of the key mechanisms of secondary injury (sub-acute phase). It includes the activation of resident cells (microglia, astrocytes), the recruitment of immune cells (macrophages and neutrophils) from the bloodstream to the site of the injury, and evident up-regulation of the NADPH oxidase (NOX) enzyme. The resident and immune cells release proinflammatory cytokines, including interleukins (IL-1β, IL-6) and tumor necrosis factor-α (TNF-α), all of which increase the extent of the inflammatory response. These events play an important role in secondary tissue damage and cell death [2,3,5,6]. During this period, the astrocytes begin to migrate out of the epicenter, producing molecules, such as proteoglycans and laminin, in the extracellular space. In the chronic phase of injury, there is continued necrosis, and demyelination in the white matter due to apoptotic oligodendrocytic death. Reactive astrocytes continue to invade the region surrounding the lesion center, leading to the formation of a cystic cavity surrounded by glial scar, which has been hypothesized to be a physical and biochemical barrier to axonal regeneration [7,8]. 

Research over the last few years has revealed numerous therapeutic approaches contributing to the modulation of the inflammation response and the reduction of the negative symptoms of a SCI [9,10,11,12]. Statins, the 3-hydroxyl-3-methylglutaryl-coenzyme A reductase inhibitors, are currently used for treatment of high cholesterol, coronary artherosclerosis, and atherosclerosis [13,14,15]. Atorvastatin (ATR), a drug with anti-inflammatory and immunomodulatory effects, has been widely studied in various ischemia-reperfusion and traumatic SCI models. When applied for several days before/after a SCI, it has been found to be neuroprotective [16,17,18,19]. It significantly reduced the release of pro-inflammatory cytokines and inhibited macrophage infiltration and microglial activation. In addition, this drug inhibited apoptosis and demyelination after traumatic injury and promoted the recovery of neurological functions [16,17]. 

Although pre- or post-long-lasting treatments with ATR have been shown to be effective in limiting pathology due to a traumatic SCI [17,18], the present study focused on treating animals in the very critical first moments after injury and on finding out whether an acute single dose of this clinically relevant drug would effectively minimize the negative impact of an acute, traumatic SCI. We found that ATR (5 mg/kg, i.p.) applied immediately after a Th9 compression favorably reduced the course of acute inflammation by decreasing the release of interleukin 1β 4 h and 1 d after a SCI. This statin significantly reduced macrophage infiltration, resident cell activation, and caspase-3 expression one day post-SCI and promoted axon outgrowth in the whole cranio-caudal extent after six weeks of survival. The mitigation of the strong inflammatory response soon after a SCI promoted regeneration at the lesion site and cranially and caudally from the epicenter and significantly improved the neurological outcome from day 30 to 42. These results strongly support the use of ATR as one of the first-line therapeutic drugs to treat an acute, traumatic SCI.

## 2. Results

### 2.1. Short-Term Survival (24 h)

#### 2.1.1. Releasing of Pro-Inflammatory Cytokine IL-1β after Spinal Cord Trauma and the Atorvastatin Treatment

**Figure 1:**
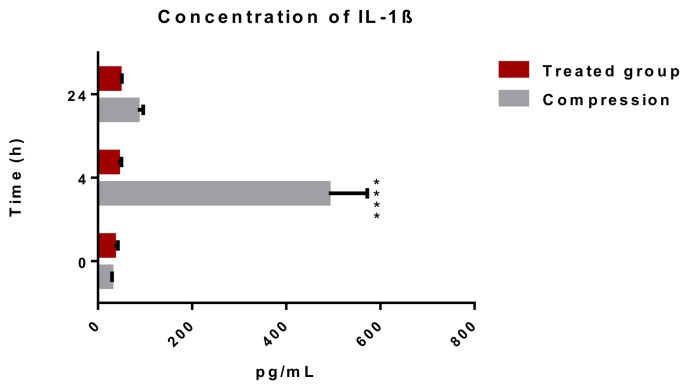
Concentration of pro-inflammatory cytokine IL-1β in the blood serum after a traumatic SCI and the ATR treatment. A significant elevation of IL-1β was noted 4 h after the SCI. ATR applied in a single dose (5 mg/kg; i.p.) immediately after the SCI reduced the release of IL-1β after 4 and 24 h. Data are the mean values of eight experiments ±SD. The results were statistically evaluated using two-way analysis of variance (ANOVA) and post hoc Tukey’s HSD test; **** *p* < 0.0001. ATR—Atorvastatin; IL-1β—interleukin 1β; SCI—spinal cord injury.

The activation of the inflammatory response after a spinal cord injury leads to the release of pro- and anti-inflammatory cytokines into the bloodstream [20]. We examined the effect of a single dose of ATR (5 mg/kg; i.p.) on the level of pro-inflammatory cytokine IL-1β in the blood serum at 4 and 24 h after a SCI. Figure 1 shows strong, up to 12-fold increase in the level of IL-1β (490.3 ± 82 pg/mL) at the acute (4 h) time point and rapid decrease in the cytokine level (84.8 ± 11.6 pg/mL) 24 h after the SCI. The concentration of IL-1β remained 2.9-fold higher after the SCI than the in naive spinal cord (29.3 ± 1.2 pg/mL). A single dose of Atorvastatin injected immediately after a Th9 compression decreased the release of IL-1β to 42.8 ± 7.3 pg/mL after 4 h. A similar level of IL-1β was maintained 24 h after the SCI and the ATR treatment (46.3 ± 4.6 pg/mL).

#### 2.1.2. Macrophage Response after a Spinal Cord Compression and the ATR Treatment

**Figure 2:**
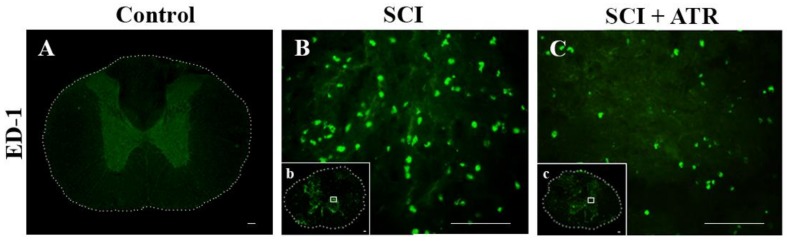

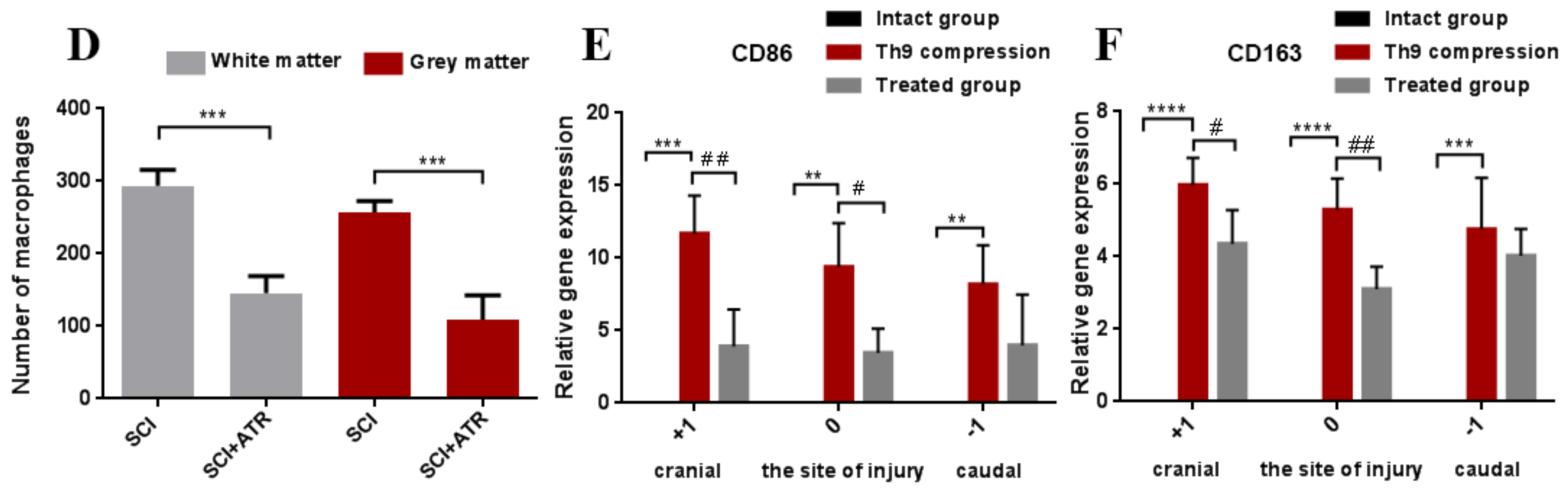
Infiltration of the macrophages in the spinal cord 24 h after a traumatic injury and the treatment with ATR (5 mg/kg; i.p.). Figures from immunohistochemical analysis show no appearance of the macrophages in the intact spinal tissue (**A**), marked macrophage infiltration at the lesion site (**B**) and a strong decrease in macrophage influx after the ATR treatment (**C**). ATR significantly reduced the number of infiltrated macrophages in the grey and white matter (**D**). CD86 mRNA (M1 macrophages) and CD163 mRNA (M2 macrophages) were confirmed by RT-PCR in the whole cranio-caudal extent of the spinal cord (**E**,**F**). Scale bars: (**A**,**b**,**c**—1000 µm; **B**,**C**—100 µm). Data are the mean values of nine experiments (4 IHC, 5 RT-PCR) ± SD. The results from the cell counting were statistically evaluated using a parametric *T*-Test and the results from the RT-PCR were evaluated with one-way ANOVA; # *p* < 0.05; ** *p* < 0.01; ## *p* < 0.01; *** *p* < 0.001; **** *p* < 0.0001. Atorvastatin—ATR; ED-1—macrophages; IHC—immunohistochemistry; SCI—spinal cord injury.

The ED-1 antibody, recognizing an intracellular antigen in activated macrophages, was used for identification of macrophages (ED-1) in immunostained cross-sections at the lesion site (Figure 2). No infiltrated ED-1 positive cells were detected in the intact spinal cord tissue; however, one day after the SCI, the macrophage infiltration was obvious (Figure 2B). Quantitative analysis revealed a higher number of ED-1 positive cells in the white matter (293 ± 22.9) than in the grey matter (257 ± 20.4). ATR significantly reduced the number of infiltrated macrophages in both the white (145 ± 40) and grey (109 ± 33) matter (Figure 2C,D).

Macrophages exhibit functional adaptability and can change phenotypes in response to stimuli [21]. One day after the SCI, both CD86 mRNA (M1 phenotype) and CD163 mRNA (M2 phenotype) were expressed at the lesion site and 1 cm cranially and caudally (Figure 2E,F). ATR significantly inhibited both the M1 and M2 phenotypes, but the decrease was more pronounced in the M1 phenotype, known to initiate a cascade of neurotoxic responses. Quantitative RT-PCR showed a significant decrease in CD86 mRNA expression at the lesion site (*p* < 0.05) and cranially from the site of the injury (*p* < 0.01) and significantly suppressed the expression of CD163 mRNA (anti-inflammatory M2 phenotype) cranially (*p* < 0.05) and at the injury site (*p* < 0.01) (Figure 2F).

#### 2.1.3. Changes in Glial Activation and Caspase-3 Activity

**Figure 3:**
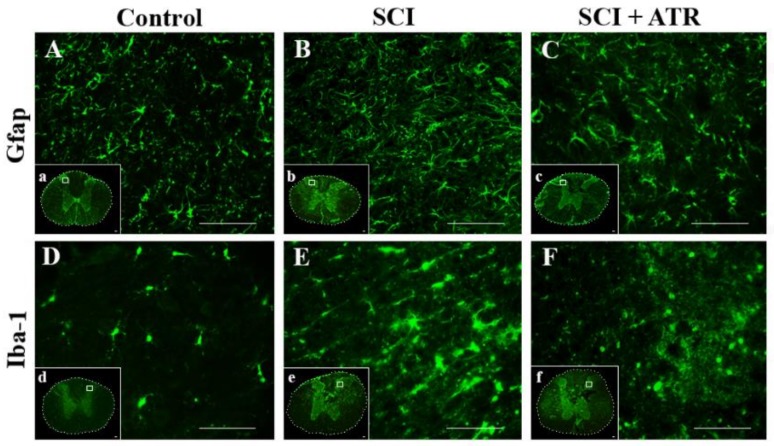
Representative images showing the activation of astrocytes and the microglia in the spinal cord after the Th9 compression and the atorvastatin treatment. Immunostaining of the astrocytes and microglial cells at the Th9 level of intact animals (**A**,**D**). Increased expression of the astrocytes (**B**) and the microglia (**E**) 24 h after the injury at the lesion site. The administration of ATR (5 mg/kg; i.p., immediately after the SCI) reduced the density of the activated astrocytes (**C**) and decreased the massive activation of the microglial cells (**F**). Scale bars: (**A**–**F**—100 µm; **a**–**f**—1000 µm). Atorvastatin—ATR; GFAP—astrocytes; Iba-1—microglia; SCI—spinal cord injury.

The GFAP antibody revealed a small stellate perikaryon of astrocytes, which had a few thin branched processes. Such morphology of GFAP-immunoreactive astrocytes was seen in the naive spinal cord (Figure 3A) and the in spinal tissue one day after the SCI. However, the activity of the astrocytes was more profound in the damaged regions within the impact area (Figure 3B). At the same time, the Iba-1 production, a marker for microglia, was strongly elevated (Figure 3E) at the site of the injury. The microglial cells were hypertrophied and massively distributed in the dorsal horn. Treatment with ATR (5 mg/kg; i.p.) decreased the extensive microglial activation (Figure 3F) and moderated the formation of reactive astrocytes at the lesion site (Figure 3C). 

**Figure 4:**
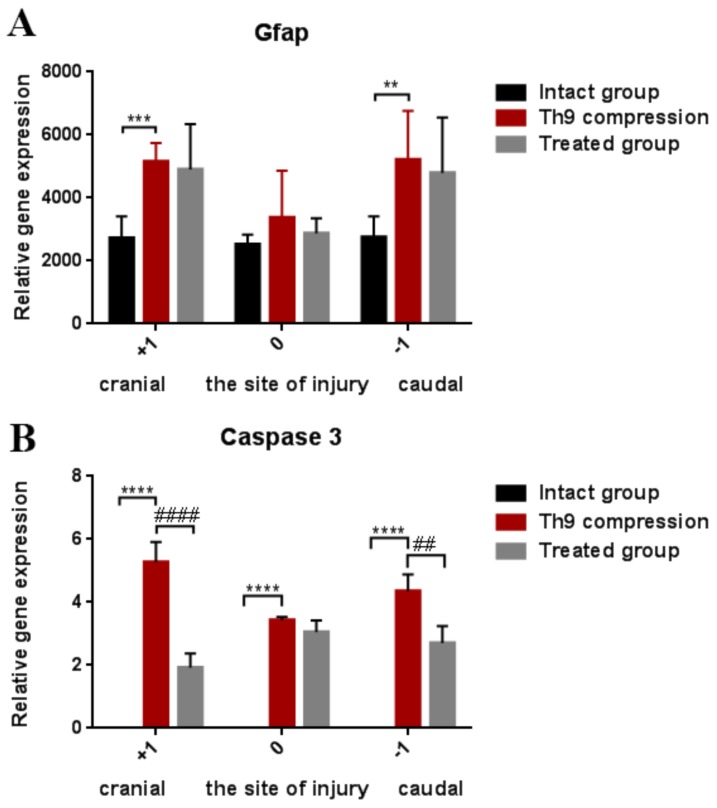
Gene expression showing astrogliosis (GFAP) and apoptosis (caspase-3) in the spinal cord 24 h after the Th9 compression and the atorvastatin (5 mg/kg; i.p.) treatment. The graphs show the relative quantities of GFAP (**A**) and caspase-3 (**B**) in rostro-caudal manner in the controls and 24 h after the SCI and SCI + ATR treatment. Data are the mean values of five experiments ±SD. The results were statistically evaluated using one-way ANOVA; ** *p* < 0.01; ## *p* < 0.01; *** *p* < 0.001; **** *p* < 0.0001; #### *p* < 0.0001. Atorvastatin—ATR; GFAP—astrocytes; SCI—spinal cord injury.

Figure 4A shows the course of astrogliosis in a 3 cm cranio-caudal extent 24 h after the SCI. The GFAP gene expression was overexpressed in the cranial (*p* < 0.001) and caudal (*p* < 0.01) segments after the SCI, but at the site of the injury this astrocyte marker was not significantly increased. GFAP mRNA expression was not markedly changed by the ATR treatment. However, compared with the controls, a moderate improvement was visible in the caudal segment (−1). 

**Figure 5:**
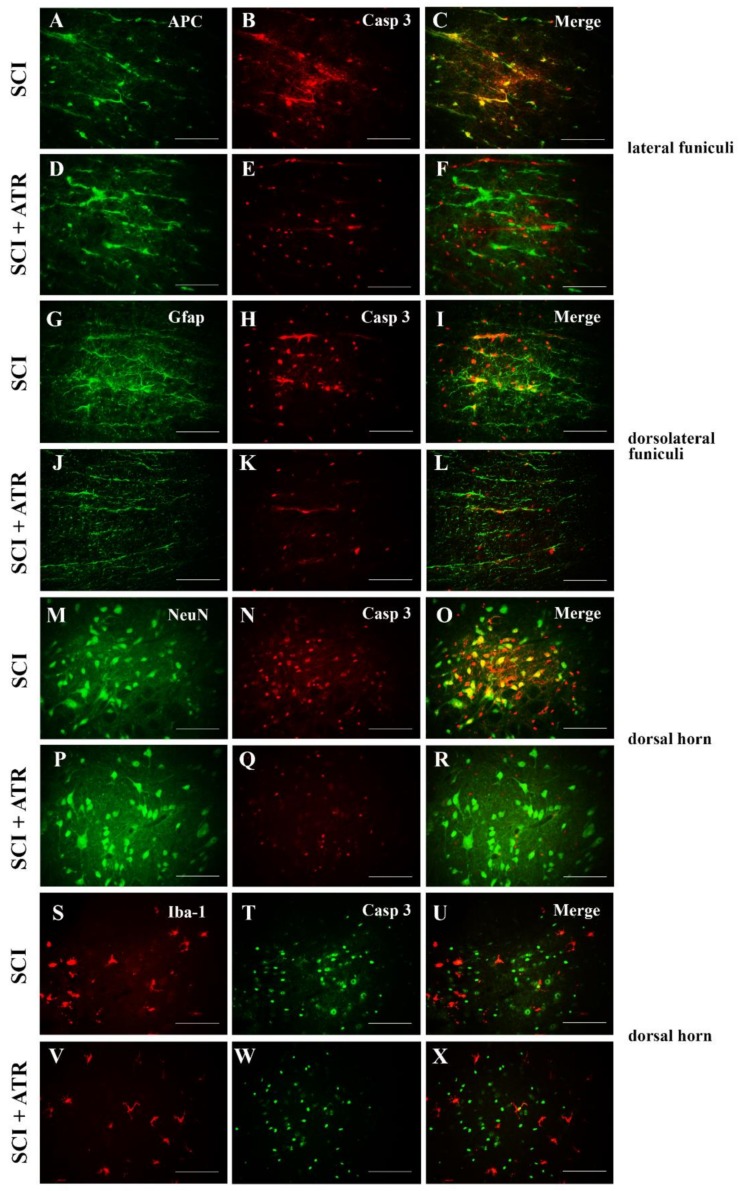
Set of microphotographs showing the immunofluorescent staining and co-localization of caspase-3 (red in B,E,H,K,N,Q; green in T,W) with APC (green), GFAP (green), NeuN (green), and Iba-1 (red) one day after the SCI and the ATR treatment. Double immunostaining demonstrates the cleavage of caspase-3 in the oligodendrocytes (**A**–**F**); astrocytes (**G**–**L**); neurons (**M**–**R**); and microglial cells (**S**–**X**) 0.5 cm caudally from the lesion site in the SCI and SCI + ATR groups. Scale bars: (**A**–**X**—100 µm). APC—adenomatous polyposis coli positive mature oligodendrocytes; ATR—Atorvastatin; Casp 3—caspase-3; GFAP—astrocytes, Iba-1—microglia; NeuN—neurons; SCI—spinal cord injury.

Caspases, including caspase-3, directly and indirectly control the changes in cells during apoptosis [22]. Strong mRNA expression of caspase-3 was confirmed one day after the Th9 compression in the whole cranio-caudal extent (Figure 4B). Atorvastatin (5 mg/kg; i.p.) significantly decreased the expression of caspase-3 mRNA in the cranial (*p* < 0.0001) and caudal (*p* < 0.01) segments. The results from the double immunostaining showed strong cleavage of caspase-3 in the oligodendrocytes, astrocytes, and neurons all around the lesion site 24 h after the Th9 compression (Figure 5). A marked reduction in apoptotic activity was noted after the ATR treatment in the oligodendrocytes and astrocytes in the dorsal and dorsolateral funiculi. Immunolabeled NeuN positive cells demonstrated a massive decrease in caspase-3 activity especially in the dorsal horns but also around the central canal. However, no cleavage of caspase-3 was observed in the microglial cells either after the SCI or after the ATR treatment at this time point. 

### 2.2. Long-Term Survival (Six Weeks)

#### 2.2.1. Regenerative Capacity of the Spinal Cord

**Figure 6:**
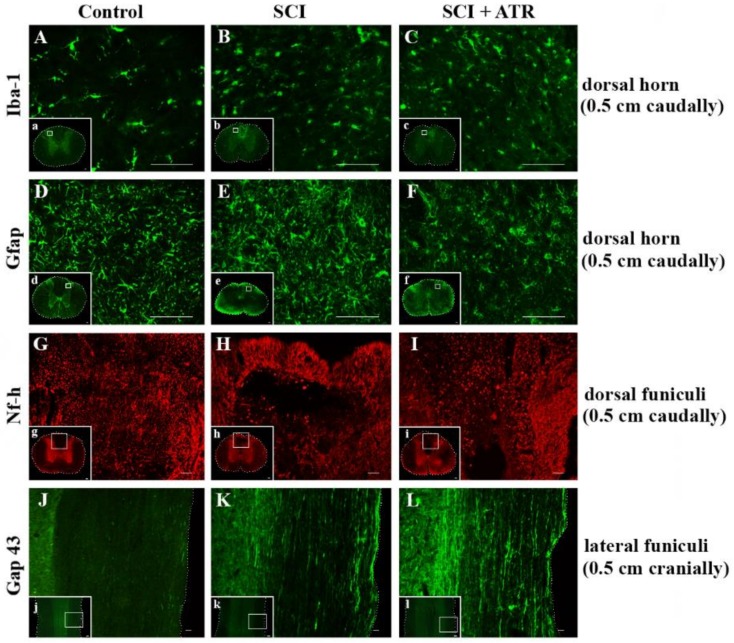
Effect of Atorvastatin on the glial cell activation and the regenerative capacity in the spinal cord six weeks after the SCI and the ATR treatment (5 mg/kg; i.p.). The microphotographs show visible changes in the activation of the microglia (green) (**A**–**C**) and astrocytes (green) (**D**–**F**) in the dorsal horn (0.5 cm caudally from the site of the injury) after the SCI and SCI + ATR. The regenerative capacity of Nf-h (red) is clearly visible in the dorsal funiculi caudally from the lesion site (**G**–**I**). Longitudinal spinal cord sections taken from the cranial segments show spontaneous axonal outgrowing (Gap43; green) after the SCI (**K**). More pronounced Gap43 immunoreactivity was visible in the ATR-treated group (**L**). Scale bars: (**A**–**F**—100 µm; **G**–**I**—200 µm; **J**–**L**—500 µm; **a**–**l**—1000 µm). ATR—Atorvastatin; Gap 43—outgrowing axons; GFAP—astrocytes; Iba-1—microglia; Nf-h—neurofilaments (heavy); SCI—spinal cord injury.

**Figure 7:**
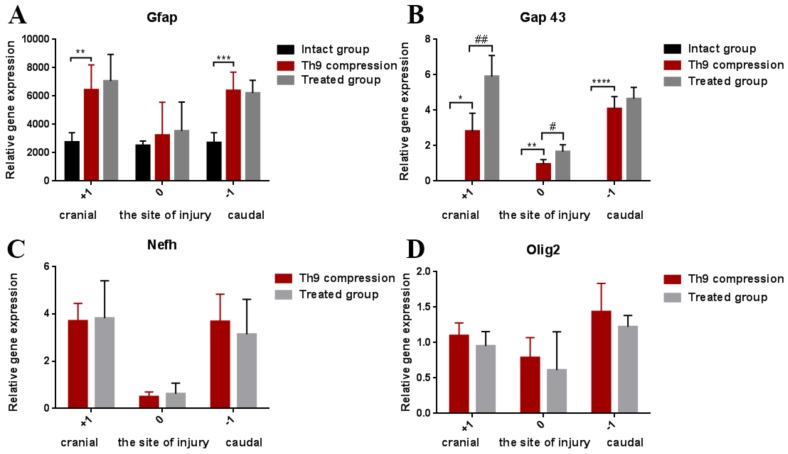
Graphs demonstrating the relative gene expression of GFAP, Gap 43, Nf-h, and Olig 2 in the spinal cord (site of the injury, cranially, and caudally) six weeks after the traumatic spinal cord injury and the ATR treatment. The lowest gene expression was observed at the lesion site. GFAP activation shows the changes in astrogliosis after the SCI and SCI + ATR (**A**). Markers of regenerative capacity (Gap 43, Nf-h, and Olig2) show a significant improvement in axonal outgrowing at the lesion site and in the cranial (+1) segment (**B**–**D**). The data are the mean values of five experiments ±SD. The results were statistically evaluated using a parametric *T*-test and one-way ANOVA; * *p* < 0.05; # *p* < 0.05; ** *p* < 0.01; ## *p* < 0.01; *** *p* < 0.001; **** *p* < 0.0001. Atorvastatin—ATR; Gap 43—outgrowing axons; GFAP—astrocytes; Nf-h—neurofilaments (heavy); Olig2—oligodendrocytes; SCI—spinal cord injury.

Six weeks after the SCI, numerous Iba-1 positive cells with rounded morphology were identified predominantly in the dorsal part of the spinal cord (Figure 6B). No obvious difference was observed visually between the SCI and ATR-treated SCI rats at six weeks in the dorsal horn (Figure 6C). As shown in Figure 6F, the GFAP immunoreactivity was markedly reduced in the ATR-treated group. Although ATR effectively reduced astrogliosis in the most affected dorsal horn of the caudal segment, the gene expression of GFAP showed no significant difference between the SCI and SCI + ATR groups in the cranial, injured, and caudal segments, each taken as a whole (Figure 7A). 

Nf-h immunoreactive axons were regularly distributed throughout the control spinal cord (Figure 6G). At six weeks, the Th9 compression resulted in massive damage to the white matter in the dorsal and lateral columns, with their partial obliteration by a cavity (Figure 6H). The ATR treatment increased the expression of neurofilaments in the dorsolateral part of the spinal cord (Figure 6I). However, compared with the SCI group, the Nf-h mRNA measured in the segments (cranial, injured, and caudal) in toto was not increased in the ATR-treated group (Figure 7C). Similar results were observed in the gene expression of Olig2 (Figure 7D).

Axonal sprouting and outgrowing in the damaged spinal cord is one of the key factors for tissue regeneration and motor function restoration. As shown in Figure 6K, spontaneous axonal overgrowing was very low six weeks post-SCI. Immufluorescence analysis of the longitudinal spinal cord sections revealed that the single dose of ATR (5 mg/kg, i.p.) strongly promoted the axons’ outgrowth (Figure 6L). This effect was visible in the whole cranio-caudal extent. The results from the RT-PCR are shown in Figure 7B. The relative gene expression of Gap43 correlated with Gap 43 immunoreactivity. The ATR-treated group confirmed a significant improvement at the site of the injury (*p* < 0.01) and in the cranial segment (*p* < 0.05). 

#### 2.2.2. Neurological Outcome

**Figure 8:**
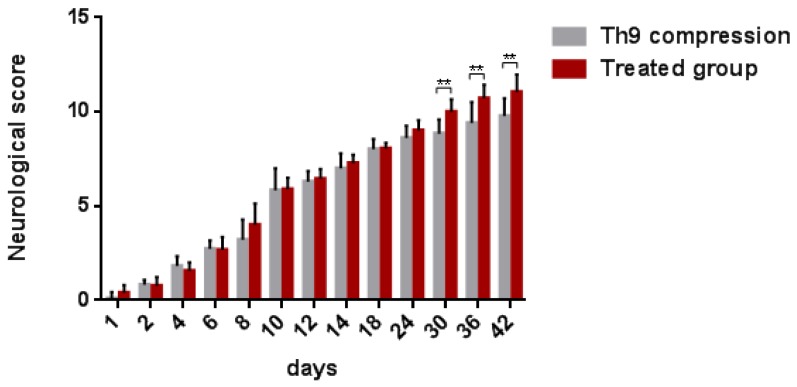
BBB scores showing the locomotor function of rats after the Th9 compression and the ATR treatment. The scoring points range from complete paraplegia (point zero) to normal hindlimb function (point 21). Data are the mean values of eighteen experiments ±SD. The results were statistically evaluated using a parametric *T*-test. Atorvastatin—ATR; BBB score—Basso-Beattie-Bresnahan score.

Functional outcome was evaluated using the Basso–Beattie–Bresnahan (BBB) locomotor rating score representing the recovery stages for rat hindlimb motor function. One day post-injury, all the animals suffered from complete paraplegia (0.25 ± 0.39) and their motor function improved spontaneously up to day 42 (six weeks) with a mean neurological score of 9.7 ± 0.93 (Figure 8). Up until day 24, the development of the neurological score did not differ between the SCI and ATR-treated groups. However, the outcome was significantly different from day 30 to 42. At the end of survival, the mean score for motor outcome reached 11.06 ± 0.91 in the ATR-treated group.

## 3. Discussion

Neuroinflammation is a complex immune response, which occurs within a short time period after SCI. It includes the activation and infiltration of numerous cell populations (astrocytes, microglia, T lymphocytes, neutrophils, and monocytes) and the release of a large number of non-cell mediators (interleukins, interferons, and TNFα) [23]. The first factors involved in the inflammatory response are pro- and anti- inflammatory cytokines and chemokines produced by endothelial cells and activated microglia [24]. In the CNS, the main pro-inflammatory cytokine is IL-1β, which is released just 2 h post-SCI [23,25]. In the present study, the levels of IL-1β in the blood serum were markedly (12-fold) elevated 4 h after the Th9 compression, but one day post-injury its concentration significantly decreased. Allan et al. [26] reported that this pro-inflammatory cytokine can directly affect glial cells, endothelial cells, and even neurons, as they all express interleukin 1 receptor type 1 (IL-1R1). In their rat SCI model, Nesic et al. [27] used infusions of IL1 antagonists 72 h after a SCI and reported markedly reduced injury-induced apoptosis, indicating that early expression of IL-1β is detrimental. It has also been shown that IL-1β promotes astrogliosis and triggers the astrocytic release of glutamate, nitric oxide, potassium, prostaglandins, cytokines, and chemokines [28], all of which were shown to be toxic to neurons at high concentrations [29,30,31]. The anti-inflammatory drug ATR applied in a single dose acutely after a Th9 compression significantly reduced the release of IL-1β.

Several studies have highlighted the therapeutic benefit of pre- and/or post-SCI application of ATR (long-lasting vs single and per-oral vs intraperitoneal). The present study produces new data showing the neuroprotective effect of a single dose of ATR applied immediately after a Th9 compression. First, ATR reduced the activation of the microglia within the most injured dorsolateral spinal cord and significantly reduced the infiltration of macrophages into the white and grey matter one day post-SCI. Papa et al. [9] showed that early microglial activation after spinal trauma is a key factor in the formation of a pathological environment, which supports injury progression. These authors demonstrated that early microglial inhibition induced a long-lasting recovery post-SCI (up to 63 days). As reported previously, microglia acquire pro-inflammatory phenotypes immediately after a SCI, which promote local inflammation and can lead to destructive (M1 phenotype) and beneficial (M2 phenotype) macrophage recruitment [9]. Based on their phenotype and activation status, macrophages may initiate the mechanisms of secondary injury or promote neuroregeneration of the spinal cord tissue [32]. One day after the SCI, we observed a significant increase in the expression of both the M1 and M2 phenotypes in each spinal cord region, but the presence of both macrophage phenotypes was not equal. The gene expression of the pro-inflammatory M1 macrophage marker was substantially higher compared with the M2 marker. ATR applied soon after the Th9 compression reduced the expression of both the M1 and M2 associated genes in all the evaluated areas, but a significant decrease was noted only in the cranial segments and at the lesion site. These segments are known to host a neuroprotective and neurotrophic environment [33]. Here, we also show that in the acute phase, ATR modulated the M1 macrophage phenotype more markedly than the M2 antigenic marker. Although the neuroprotective effect of ATR on macrophage infiltration has been shown in various SCI models, the precise time point of microglia/macrophage polarization and the factors controlling their shifts are still unclear. Khayrullina et al. [34] reported that acute inhibition of NOX2 (the enzyme that is a primary source of reactive oxygen species) with the specific inhibitor, gp91ds-tat, shifted microglial/macrophage polarization toward the M2 marker at seven days post-SCI. Although single acute gp91ds-tat treatment did not induce inflammatory effects beyond seven days, functional improvement continued through at least 28 days, indicating the importance of this single acute intervention. Bermudez et al. [35] also studied the correlation between NOX expression and microglial/macrophage polarization in a mouse SCI model. These authors confirmed that the expression of two NOX isoforms (NOX2 and NOX4) was temporally and polarization related, with an M1 preference for NOX2 acutely and NOX4 chronically; the M2 polarization marker was identified at acute time points only. While both the M1 and M2 microglia/macrophages express NOX isoforms, there is an influence of NOX on polarization. These data confirmed that NOX enzyme inhibition can alter the polarization status, which plays a significant role in the functional outcome. The regulation of the polarization of the M1/M2 macrophages after a SCI may also be related to neurotrophin-3 (NT-3) [36]. Electroacupuncture at GV acupuncture points, which can be used as an adjuvant therapy for a SCI, inhibited the proportion of M1 macrophages and proinflammatory cytokines, but at the other site it upregulated the M2 marker and NT-3 expression. Although we did not study the antioxidant effect of ATR, it seems that early ATR treatment, preferentially decreasing the M1 expression in segments with neuroprotective/neurotrophic environment could be involved in the oxidative response, thus reflecting the redox state of the lesion microenvironment. Pre-ischemic administration of ATR in a spinal cord ischemia-reperfusion model of a rabbit inhibited the depletion of antioxidative enzymes, reduced lipid peroxidation, and improved the extent of locomotor recovery [37]. In addition, increased levels of myeloperoxidase (MPO) were detected in the spinal cord following ischemia/reperfusion, suggesting leukocyte infiltration into the spinal cord. The authors have shown that ATR pre-treatment prevented MPO elevation. Secondly, astrocytes play a role in the mechanical and metabolic support of neurons. After spinal cord trauma, a select population of astrocytes, known as reactive astrocytes, upregulates the expression of intermediate filament proteins, proteoglycans, and other molecules and contributes to the inhibition of axonal outgrowth and glial scar formation [38,39,40]. One day after Th9 compression, we detected significant upregulation of GFAP mRNA in the areas surrounding the lesion site. Over time, the GFAP mRNA was only moderately reactivated at six weeks of survival. This finding might be associated with the regulation of the ion concentration in the extracellular space, the modulation of synaptic transmission, and the repair of the spinal parenchyma through the formation of a glial scar in the later post-SCI period [41]. Pannu et al. [16,17] injected ATR (5 mg/kg) through the whole period of survival, and they demonstrated a significant reduction of astrocyte activation. A single dose of ATR (5 mg/kg; i.p.) injected immediately after the SCI modulated the acute immune response, but we did not see its significant short- or long-lasting effect. It is also evident that although inflammation plays a very important role in the development of a glial scar, paradoxically, astrocyte activation by IL-1β can exert neuroprotective effects by stimulating the repair of the blood–brain barrier and decreasing its permeability [25]. Recently published data show that specific parts of the astrocytic scar have a supportive function for axon regeneration following a SCI [42] and might be induced by activated microglia [43]. Thirdly, early application of ATR could be crucial for limiting the secondary injury cascade after a SCI. One day after the Th9 compression, we noticed a sharp caspase-3 mRNA expression throughout the whole injured area. Moreover, the double immunostaining of caspase-3 with NeuN, APC, or GFAP showed marked apoptotic activity of these cells around the lesion site. Treatment with ATR, applied immediately after the Th9 compression, significantly reduced the expression of apoptotic markers. Immunohistochemical analysis showed the strong effect of the treatment on the reduction of caspase-3 cleavage in the oligodendrocytes, neurons, and astrocytes both cranially and caudally from the epicenter. Several recent studies have demonstrated the beneficial effect of ATR on the functional outcome through apoptosis inhibition. Gao et al. [18] showed that two doses of ATR (5 mg/kg; i.p.) injected one and two days after a weight-drop spinal cord injury significantly reduced caspase-3 and caspase-9 expression and activated autophagy, which was conducive to the recovery of neurological functions at seven days post-injury. Intraperitoneal injection of this drug was also effective in preventing early apoptosis at the lesion site within 2 h post-SCI administration [19]. ATR-treated rats showed a significant decrease in caspase-3 activity and a decrease in the number of TUNEL-positive cells at the injury site 4 h post-SCI, followed by an improvement in locomotion at four weeks post-SCI. While the treatment with ATR prevented excessive caspase-3 activation, it also inhibited the massive extension of the inflammatory response. Our results indicate that early inhibition of caspase-3 activity in the oligodendrocytes, neurons, and astrocytes may have a role in mitigating neurodegeneration, as well as in the remarkable functional outcome seen from day 30 to 42 after the Th9 compression. Gao et al. [44] also pointed out the positive effect of the early inhibition of programmed cell death. After Simvastatin injection (two doses, 10 mg/kg; i.p.), they observed a significant improvement in locomotor functional recovery seven weeks after a weight-drop SCI. It has been suggested that oligodendrocyte apoptosis contributes to chronic demyelination of spared axons and exacerbates the extent of the injury, eventually leading to permanent functional deficits [45]. Lee et al. [45] demonstrated that preventing oligodendrocyte cell death improved the neurological score for 35 days after a SCI in mice. 

We suggest that the neuroprotective effect of ATR registered soon after a SCI could be crucial for improving tissue regeneration and the functional outcome. Markers of axonal outgrowing (Gap 43) quantified by means of the RT-PCR showed improvement in each evaluated segment. Gap-43 immunoreactive axons were distinctly visible throughout the whole cranio-caudal extent, but the most extensive Gap-43 immunoreactive axons were seen in the lateral funiculi. Similarly, a very strong regenerative response was detected in the expression of the neurofilaments. Neurofilaments are particularly abundant in large myelinated axons and are essential for axon radial growth and caliber maintenance during development [46,47]. We detected a loss of neurofilament-positive axons six weeks after the Th9 compression in areas infiltrated with Iba-1 positive cells. Massive regeneration of neurofilament immunoreactive axons was detected in the injured dorsolateral funiculi after the ATR treatment, although neurofilament mRNA measured in toto did not show a significant improvement. Wang et al. [48] have reported that neurofilament gene transcriptional regulation is crucial for neurofilament expression predominantly in axonal regeneration. We have also recently pointed out the role of neurofilaments in modulating spinal microcircuits leading to a better functional outcome [49]. Although the Olig2 transcription factor, an essential regulator of oligodendrocyte development, has been seen to improve locomotor recovery and enhance myelination in a rat contusive spinal cord injury model [50], we did not see any neuroprotective effect of the ATR treatment in Olig2 mRNA. 

This study underscores the protective effect of ATR applied immediately post-SCI. The results suggest that ATR, even in a single dose, has important neuroprotective potential. However, a set of research problems dealing with practical ATR application in a traumatic SCI injury currently remains unexplored.

## 4. Materials and Methods 

### 4.1. Experimental Animals and Surgical Procedure 

A total of 48 adult female Wistar rats (weighing 250–300 g) were used in the experiment. The rats were housed 5 per cage on a 12-h dark/light cycle in a temperature- and humidity-controlled environment. Female rats were used in order to relieve serious urinary tract complications after SCI, such as hematuria (hemorrhagic cystitis). Ferrero. S.L. et al. [51] showed that after contusion injury, hematuria duration was significantly longer in males compared with females, despite similar bladder reflex onset times. All procedures were carried out in accordance with the protocols approved by the State Veterinary and Food Administration in Bratislava (decision No. 4434/16-221/3), as well as by the Animal Use Committee at the Institute of Neurobiology, Slovak Academy of Sciences, and in accordance with the EC Council Directive (2010/63/EU) regarding the use of animals in research. All efforts were made to minimize the number of rats and their suffering. 

The animals were divided into five experimental groups as follows: (1) control animals (*n* = 6); (2–3) rats subjected to the Th9 compression surviving for 24 h (*n =* 12) and six weeks (*n =* 9); and (4–5) rats after the SCI treated with post-surgical Atorvastatin administration (5 mg/kg, i.p.; Fluka by Sigma Aldrich, St. Louis, MO, USA) surviving for 24 h (*n =* 12) and six weeks (*n =* 9). 

The surgical procedure was performed under isoflurane anesthesia (2–4%; AbbVie, BA, Slovak Republic; in 1.5–2.0 L/min oxygen), delivered by mask. After laminectomy at the Th9 segment, SCI was induced using a compression device with a weight of 40 g/15 min. The body temperature was maintained at 37 °C during the whole surgical procedure. After the Th9 compression, the rats received the antibiotic drug Amoksiklav (Sandoz Pharmaceuticals, Ljubljana, Slovenia; 30 mg/kg, i.m.) and the analgesic drug Novasul (Richterpharma, Wels, Austria; 2 mL/kg, i.m.) for three days. The Atorvastatin group of animals was treated with a single dose of Atorvastatin (5 mg/kg, i.p.), applied immediately after the SCI. The animals were housed in separated cages to recover with access to food and water ad libitum. The bladder expression of the rats after the SCI was required twice a day until the bladder reflex was restored (14–18 days).

### 4.2. Enzyme-Linked Immunosorbent Assay (ELISA)

Blood from the rats’ tail vein (250–300 µL) was taken before the SCI, 4 and 24 h after trauma (4 animals/group). Each sample was centrifuged 10 min at 10,000 rpm to obtain the blood serum. The supernatants were kept at −70 °C until the ELISA procedure was performed. The level of IL-1β was measured using a Rat IL-1β ELISA Kit (Thermo Fisher Scientific, Waltham, MA, USA) according to the manufacturer’s instructions.

### 4.3. Immunohistochemistry

At the end of the experiments, the rats (*n* = 16) were deeply anesthetized with thiopental (Valeant Czech Pharma s.r.o., Prague, Czech Republic; 50 mg/kg, i.p.) and perfused transcardially with 300 mL saline followed by 300 mL 4% paraformaldehyde in 0.1 M phosphate-buffered saline (PBS; pH 7.4, Sigma-Aldrich, St. Louis, MO, USA). Spinal cord blocks (0.5 cm cranially and caudally from the epicenter of the injury and the site of the injury) were post-fixed in the same fixative overnight and subsequently cryopreserved in a solution of 30% sucrose in PBS at 4 °C for 2 days. Afterwards, each tissue block was cut into transverse serial and longitudinal sections (25 µm thick) on a cryostat (Leica CM1850, Wetzlar, Germany). The sections were washed (3 × 10 min) in PBS with 0.3% Triton X-100 (Sigma-Aldrich, St. Louis, MO, USA) and blocked for 30 min at room temperature in a PBS solution containing 5% normal goat, rabbit, or donkey serum and 0.3% Triton X-100. For the processing of macrophages, microglia, astrocytes, neurofilaments, and outgrowing axons, the sections were incubated overnight at 4 °C with the following primary antibodies: ED-1 (mouse, 1:400 Biorad, Hercules, CA, USA), IBA-1 (goat, 1:1000 abcam, Cambridge, UK), GFAP (mouse, 1:600 Burlington, MA, USA), Nf-h (mouse, 1:1000 Danvers, MA, USA), and Gap43 (mouse, 1:500 abcam, Cambridge, UK). For the identification of caspase-3-expressing cells, double immunostaining was used. Anti-caspase-3 antibody (rabbit, 1:500 abcam, Cambridge, UK) was incubated overnight at 4 °C in combination with neuronal specific NeuN (mouse, 1:1000 abcam, Cambridge, UK), APC (mouse, 1:200 Millipore, Darmstadt, Germany), IBA-1, and GFAP antibodies. After washing (3 × 10 min) in PBS with 0.3% Triton X-100, the sections were incubated in the following secondary antibodies: FITC goat anti-mouse IgG (1:200); FITC rabbit anti-goat IgG (1:125); RRX goat anti-mouse IgG (1:600) (Jackson Immunoresearch, West Grove, PA, USA); AF donkey anti-rabbit IgG (1:500); AF donkey anti-goat IgG (1:500) (Thermo Fisher Scientific, Waltham, MA, USA) for 2 h at room temperature. Immunolabeled spinal cord sections were mounted with Prolong (Invitrogen by Thermo Fisher Scientific, Carlsbad, CA, USA). Images were captured using an Olympus BX51 (Tokyo, Japan) fluorescent microscope.

### 4.4. Cell Counting

The ED-1 positive cells were counted using ImageJ 1.47 software (Wayne Rasband, National Institutes of Health, Bethesda, MD, USA). The number of immunostained macrophages (ED-1) was quantified at the lesion site from 25 µm thick transverse sections. The ED-1 positive cells were counted in the grey and white matter from 20 immunofluorescently labeled sections.

### 4.5. Tissue Processing for RT-PCR

Total RNA from the Th7-Th10 spinal cord (*n* = 20) was obtained using a Trizol Reagent (Thermo Fisher Scientific, Waltham, MA, USA) according to the manufacturer’s instructions. The concentration of RNA in each sample was measured using NanoDrop 2000 c (Thermo Fisher Scientific, Waltham, MA, USA). The isolated RNA (2000 ng/µL) was reverse transcribed into cDNA in duplicates using a High-capacity cDNA Reverse Transcription Kit (AB applied biosystems by Thermo Fisher Scientific, Waltham, MA, USA) and a T1000™ Thermal Cycler (Bio-Rad, Hercules, CA, USA). cDNA samples (20 µL) were stored at −20 °C until real-time PCR was performed. 

Immediately before the RT-PCR reaction, the cDNA samples were diluted with 80 µL nuclease-free water (Thermo Fisher Scientific, Waltham, MA, USA). The amplification of cDNA was performed using the CFX96™ Real-Time System (Bio-Rad, Hercules, CA, USA). The reaction mixture (20 µL) was composed of 10 µL TaqMan^®^ Gene Expression MasterMix (AB applied biosystems by Thermo Fisher Scientific, Waltham, MA, USA), 1 µL TaqMan^®^ Gene Expression Assays (AB applied biosystems by Thermo Fisher Scientific, Waltham, MA, USA)—βactin (Rn00667869); Gap43 (Rn01474579); Cd163 (Rn1492519); Cd86 (Rn00571654); GFAP (Rn00566603); Olig2 (Rn01767116); Casp3 (Rn00563902); Nf-h (Rn00709325), 4 µL nuclease-free water and 5 µL of cDNA sample. The amplifications were run under the following conditions: 10 min at 95 °C followed by 50 cycles of 15 s/95 °C and 1 min/60 °C. Gene expression was normalized with β-actin (reference gene). As a calibration product, samples from previous reactions were used. The relative quantity of gene expression was assigned using Bio-Rad CFX Manager. For statistical analyses, GraphPad Prism version 6.01 (La Jolla, CA, USA) was used.

### 4.6. Behavioral Assessment

The neurological outcome of animals was tested using the Basso–Beattie–Bresnahan (BBB) locomotor rating scale ranging from 0 (complete paralysis) to 21 points (normal movement with trunk stability) [52]. Each rat was scored in an open field for a period of 5 min by two examiners. The locomotor assessment was carried out one day after the SCI, every other day for the first two weeks, and then once a week until six weeks post-injury.

### 4.7. Statistical Analysis

The data from the BBB locomotor test was analyzed using Student’s parametric *T*-Test. One-way analysis of variance (ANOVA) was used to determine the statistical significance (*p* < 0.05) of the differences between the compression group, ATR-treated group, and control group. All these data were analyzed using GraphPad Prism version 6.01 (La Jolla, CA, USA) and were expressed as mean values with standard deviation (SD). The data from the ELISA were analyzed using two-way ANOVA, in which the time after the spinal cord injury was the first independent variable and with or without ATR was the second independent variable. These data were analyzed using StatSoft, Inc. (2013), version 12. 

## 5. Conclusions

Atorvastatin (5 mg/kg, i.p.) injected in a single dose immediately after spinal cord trauma, significantly reduced the early inflammatory response and sharply decreased the expression of caspase-3 in neurons, astrocytes, and oligodendrocytes 24 h post-SCI. Acute ATR treatment effectively prevented the excessive infiltration of destructive M1 macrophages around the lesion site, while the infiltration of beneficial M2 macrophages was less affected. This statin significantly improved the regeneration capacity of the injured tissue at six weeks, leading to the promotion of axonal outgrowth and increasing neurofilament expression. We can conclude that the early modulation of the inflammatory response via effects on the M1/M2 macrophages and the inhibition of caspase-3 expression strongly promote tissue regeneration in later periods.
